# Flame (v2.0): advanced integration and interpretation of functional enrichment results from multiple sources

**DOI:** 10.1093/bioinformatics/btad490

**Published:** 2023-08-04

**Authors:** Evangelos Karatzas, Fotis A Baltoumas, Eleni Aplakidou, Panagiota I Kontou, Panos Stathopoulos, Leonidas Stefanis, Pantelis G Bagos, Georgios A Pavlopoulos

**Affiliations:** Institute for Fundamental Biomedical Research, BSRC “Alexander Fleming”, Vari (Athens), 16672, Greece; Institute for Fundamental Biomedical Research, BSRC “Alexander Fleming”, Vari (Athens), 16672, Greece; Institute for Fundamental Biomedical Research, BSRC “Alexander Fleming”, Vari (Athens), 16672, Greece; Department of Mathematics, University of Thessaly, Lamia, 35100, Greece; Department of Computer Science and Biomedical Informatics, University of Thessaly, Lamia, 35131, Greece; 1st Department of Neurology, Eginition Hospital, Athens, 11528, Greece; School of Medicine, National and Kapodistrian University of Athens, Athens, 11527, Greece; 1st Department of Neurology, Eginition Hospital, Athens, 11528, Greece; Department of Computer Science and Biomedical Informatics, University of Thessaly, Lamia, 35131, Greece; Institute for Fundamental Biomedical Research, BSRC “Alexander Fleming”, Vari (Athens), 16672, Greece; Center of Basic Research, Biomedical Research Foundation of the Academy of Athens, Athens, 11527, Greece; Hellenic Army Academy, Vari, 16673, Greece

## Abstract

Summary: Functional enrichment is the process of identifying implicated functional terms from a given input list of genes or proteins. In this article, we present Flame (v2.0), a web tool which offers a combinatorial approach through merging and visualizing results from widely used functional enrichment applications while also allowing various flexible input options. In this version, Flame utilizes the aGOtool, g: Profiler, WebGestalt, and Enrichr pipelines and presents their outputs separately or in combination following a visual analytics approach. For intuitive representations and easier interpretation, it uses interactive plots such as parameterizable networks, heatmaps, barcharts, and scatter plots. Users can also: (i) handle multiple protein/gene lists and analyse union and intersection sets simultaneously through interactive UpSet plots, (ii) automatically extract genes and proteins from free text through text-mining and Named Entity Recognition (NER) techniques, (iii) upload single nucleotide polymorphisms (SNPs) and extract their relative genes, or (iv) analyse multiple lists of differentially expressed proteins/genes after selecting them interactively from a parameterizable volcano plot. Compared to the previous version of 197 supported organisms, Flame (v2.0) currently allows enrichment for 14 436 organisms.

**Availability and implementation:**

Web Application: http://flame.pavlopouloslab.info. Code: https://github.com/PavlopoulosLab/Flame. Docker: https://hub.docker.com/r/pavlopouloslab/flame.

## 1 Introduction

Functional enrichment is the process of identifying biological functions, pathways, diseases, or phenotypes where groups of genes or proteins are involved. To this end, several applications have been developed (van den Berg *et al.*, 2009, Huang *et al.* 2009, Maleki *et al.* 2020, Chicco and Jurman 2022, Wijesooriya *et al.* 2022). DAVID (Sherman *et al.* 2022), Panther (Mi *et al.* 2021), Metascape (Zhou *et al.* 2019), WebGestalt (Wang *et al.* 2017), Enrichr (Xie *et al.* 2021), aGOtool (Schölz *et al.* 2015), AllEnricher (Zhang *et al.* 2020), GO-Elite (Zambon *et al.* 2012), MSigDB (Liberzon *et al.* 2011), WEGO (Ye *et al.* 2018), KOBAS (Bu *et al.* 2021), clusterProfiler (Yu *et al.* 2012), modEnrichr (Kuleshov *et al.* 2019), agriGO (Tian *et al.* 2017), DOSE (Yu *et al.* 2015), GeneTrail (Gerstner *et al.* 2020), GOrilla (Eden *et al.* 2009), ToppGene (Chen *et al.* 2009), PANGEA (Hu *et al.* 2023), and Enrichr-KG (Evangelista *et al.* 2023) are a few of the widely used applications which are offered either as web tools or as software packages while others such as BiNGO (Maere *et al.* 2005), stringApp (Doncheva *et al.* 2023), or ClueGO (Bindea *et al.* 2009) are offered as plugins (Saito *et al.* 2012) in larger frameworks such as Cytoscape (Otasek *et al.* 2019).

While this variety of applications allows focusing on different aspects such as enriching for (i) biological pathways [e.g. KEGG (Kanehisa and Goto 2000), WikiPathways (Slenter *et al.* 2018), Reactome (Fabregat *et al.* 2018)], (ii) Gene Ontology [GO (Aleksander *et al*. 2023)] terms such as biological processes, molecular functions and cellular components, (iii) diseases [e.g. OMIM (Amberger *et al.* 2015), DisGeNet (Piñero *et al.* 2020)], (iv) protein complexes [e.g. CORUM (Giurgiu *et al.* 2019)], (v) protein domains [e.g. Pfam (Finn *et al.* 2016)], (vi) phenotypes [e.g. HPO (Robinson *et al.* 2008)], or (vii) regulatory motifs [e.g. TRANSFAC (Matys *et al.* 2003), miRTarBase (Huang *et al.* 2020)], an obvious selection of the right tool to cover certain needs is most of the times not straightforward. Moreover, while most of these tools come with a simple interface, little emphasis has been given on the visualization and combination of results, id conversion, parameter selection, result prioritization, record association, support of multiple lists and versatile input options. Therefore, usage and interpretation of results in a more streamlined and combinatorial aspect still remain difficult tasks for the average user.

In this article, we present Flame (v2.0), an advanced service for addressing the aforementioned issues. Flame focuses on usage simplicity, offering many degrees of freedom regarding importing and handling multiple lists, running advanced state-of-the-art functional enrichment analysis pipelines as well as combining and prioritizing results through advanced interactive visualizations. Flame is able to report results as lists but also associate them at a network level (Koutrouli *et al.* 2020), while it offers major integration of resources (Baltoumas *et al.* 2021a) such as aGOtool, g: Profiler, WebGestalt, Enrichr, and STRING (Szklarczyk *et al.* 2023); thus referring to their broad audience while taking full advantage of their strengths.

## 2 Methods and results

### 2.1 Input options

**Figure 1.**
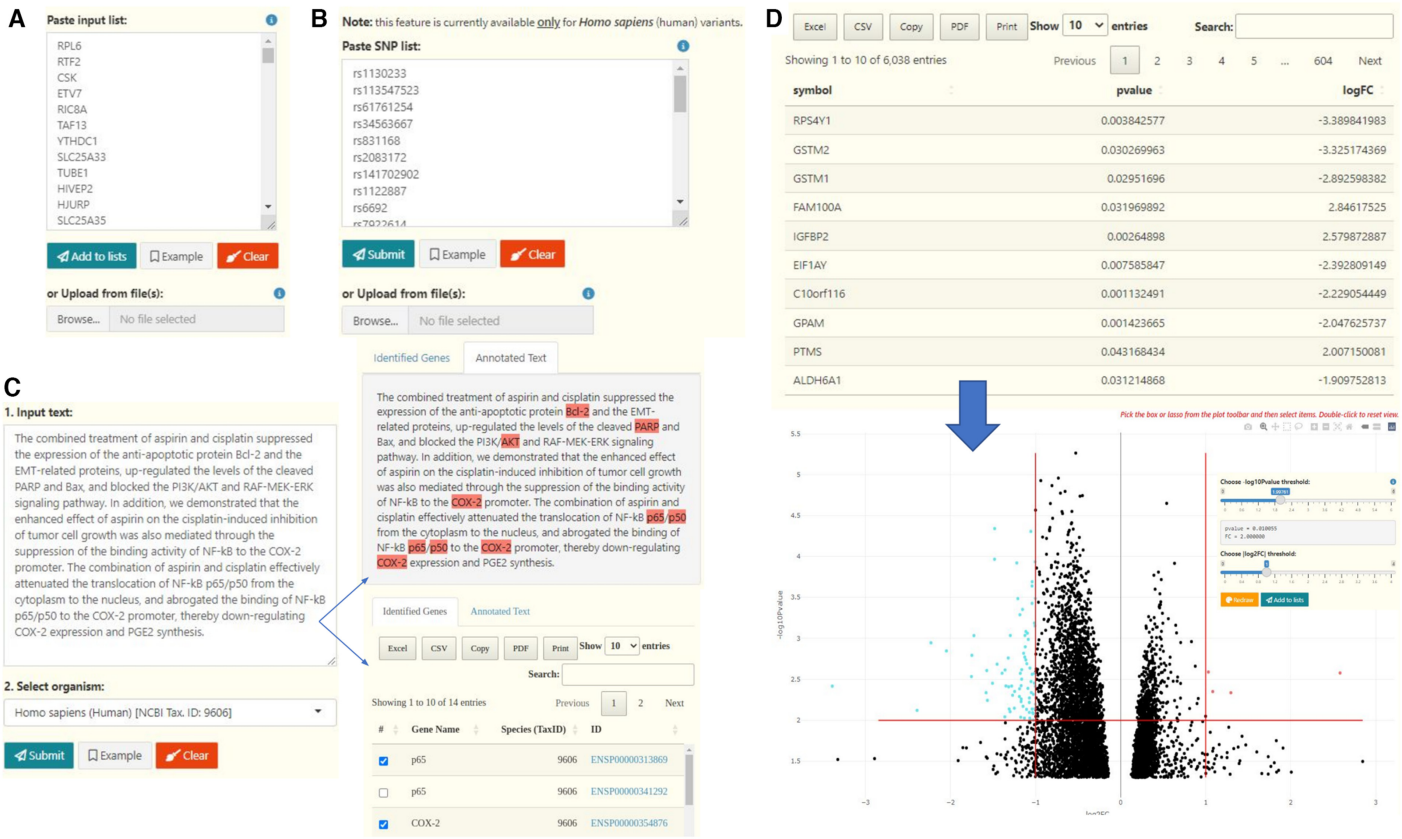
Flame input options. (A) A simple gene list using any of the widely used identifiers (e.g. HUGO names, ENSEMBL, Entrez, UniProt). (B) A list with Human SNPs. (C) A free text which can be further mined for genes and proteins using NER for an organism of preference. The identified genes and proteins can be then used for functional enrichment analysis. (D) Input of gene expression data (gene name, *P*-value, logFC) to generate a Volcano plot. Users can manually apply thresholds on the *P*-value and the FC axes to define the differentially over- and under-expressed genes of significance and interactively select them from the plot for further analysis

Compared to its predecessor (Thanati *et al.* 2021), Flame (v2.0) comes with a variety of newly introduced input options (Fig. 1). These are mainly: (i) support of simple gene lists, (ii) support of variants and polymorphisms, (iii) support of text-mining and Named Entity Recognition (NER) techniques to process free text and mine it for genes and proteins, and (iv) support of gene expression data derived from experiments.

***Gene lists***: Similarly to Flame v1.0, users can directly paste or upload multiple lists by using a plethora of gene identifiers or chromosomal intervals. Gene and protein names can be imported in the Ensembl (Cunningham *et al.* 2022), Entrez (Sayers *et al.* 2022), UniProt (UniProt Consortium 2021), RefSeq (Li *et al.* 2021), EMBL (Arita *et al.* 2021), ChEMBL (Mendez *et al.* 2019), and WikiGene (Maier *et al.* 2005) namespaces, or be later converted into another namespace during the functional enrichment analysis procedure.

***Variants and Polymorphisms***: In addition to directly uploading gene lists, Flame is capable of parsing and annotating lists of single nucleotide polymorphisms (SNPs), through the “SNP” input option. The input in this case is a list (whitespace or comma-delimited) of SNP rs-codes in the dbSNP (Sherry *et al.* 2001) format. The ids are mapped to their corresponding genes and associated Ensembl identifiers through the g: SNPense functionality of g: Profiler and are then searched against dbSNP to retrieve additional metadata, including the chromosome number, strand, genomic coordinates, and the variant’s reported effects. The annotated SNPs can be downloaded, while their associated genes can be added to the list of Flame inputs, using either their Entrez gene name or their Ensembl ids. Since dbSNP has dropped support for nonhuman variants in recent versions, this functionality is currently available only for *Homo sapiens* (human).

***Text-Mining and NER***: In this version, users can provide free text and utilize the EXTRACT (Pafilis *et al.* 2016) tagger to perform Named Entity Recognition (NER) to mine it for genes and proteins for an organism of preference. Upon completion, Flame will report the identified genes/proteins in an interactive and easy-to-filter table as well as an annotated text with the identified terms highlighted. In mouse-hover, a popup window will appear, providing information about the entity as well as direct links to the STRING and Ensembl databases. The annotated terms can be added to the list of Flame inputs, using their associated Ensembl ids. This functionality is supported for all currently available organisms in Flame (∼14 000 taxa). To this end, it is worth mentioning that the EXTRACT tagger is already being used by a plethora of established text-mining based applications (Pletscher-Frankild *et al.* 2015, Baltoumas *et al.* 2021b, Zafeiropoulos *et al.* 2022, Szklarczyk *et al.* 2023).

***Gene Expression data***: Defining the significant differentially over- and under-expressed genes is most times an empirical task performed by experimentalists, who usually apply thresholds within an accepted range for parameters such as the fold change (e.g. absolute FC > 2) and the statistical significance (e.g. *P*-value < 0.05). Therefore, slightly tuning the parameters may result in a more complete biological story. For this purpose, Flame offers the ability to upload expression data in tab delimited or comma separated format (gene name—log fold change—statistical significance) and interactively select sets of genes on a generated Volcano plot. Users can adjust both the *P*-value and FC thresholds and redraw the plot, highlighting the overexpressed genes in red and the under-expressed genes in blue. Following, users may apply a selection lasso or rectangle to mark gene sets of interest directly on the Volcano plot, and append them to the Flame lists which can then be further processed and enriched.

### 2.2 Integration and functional enrichment for multiple organisms from various sources

Due to the lack of standards in the field (Wijesooriya *et al.* 2022), functional enrichment applications often differ on the databases they support, the way of processing data, the organisms they cover, their ranking and scoring schemes, their reporting and visualizations as well as the input options they offer. While such differences may hinder usage, a combination of such tools as a means of complementing each other’s weaknesses is always an option. However, familiarity with each tool of choice is necessary but often a time consuming process. In Flame (v2.0), we overcome this issue by bringing together four established applications, namely *aGOtool, gProfiler, WebGestalt*, and *enrichR* under the same interface and making them available, in a consistent and unified manner. These four tools were selected based on the following criteria: (i) availability of programmatic access, either in the form of a package or through an Application Programming Interface (API), (ii) the number and diversity of supported databases for enrichment, (iii) the number of supported organisms, and (iv) the ability to handle large requests.

In detail, aGOtool (and subsequently Flame) currently supports 14 436 organisms corresponding to UniProt reference proteomes, including animals (mammals, insects, fish, etc.), plants, fungi, protozoa and bacteria. The tool offers functional enrichment for biological processes, molecular functions, and cellular components through Gene Ontology, pathway enrichment through KEGG, Reactome and WikiPathways, disease enrichment through the Disease Ontology (Schriml *et al.* 2019), annotation enrichment for proteins through UniProt, Pfam and InterPro (Blum *et al.* 2021) as well as tissue enrichment through the Brenda Tissue Ontology (Gremse *et al.* 2011). It also offers literature enrichment through PubMed. Regardless of the input identifiers, gene/protein names are converted to the Ensembl protein namespace (ENSP) through the STRING API before the enrichment process. The ENSP namespace returns the most accurate results for the aGOtool enrichment pipeline.

While using the g: Profiler pipeline, extra features such as enriching for tissues through the Human protein Atlas (HPA) (Karlsson *et al.* 2021), protein annotations through CORUM, phenotypes through the Human Phenotype Ontology (HPO) (Robinson *et al.* 2008), or DNA regulatory motifs through TRANSFAC and miRTarBase are made available. It is worth mentioning that g: Profiler comes with an advanced id conversion function which enables namespace and orthology conversion for 821 organisms, while it provides great flexibility on the identifiers which it accepts as input.

When opting for WebGestalt, users can further enrich their lists for OMIM, DisGeNet and GLAD4U (Jourquin *et al.* 2012) diseases, PANTHER pathways (Mi *et al.* 2021) as well as DrugBank and GLAD4U drug annotations (Wishart *et al.* 2018) while genes/proteins are automatically converted to Entrez accession identifiers. Currently WebGestalt supports twelve organisms, namely, *Homo sapiens* (human), *Mus musculus* (mouse), *Rattus norvegicus* (rat), *Bos taurus* (bovine), *Arabidopsis thaliana* (thale cress), *Drosophila melanogaster* (fruit fly), *Caenorhabditis elegans* (nematode worm), *Saccharomyces cerevisiae* (yeast)*, Danio rerio* (zebrafish), *Sus scrofa* (pig), *Canis lupus familiaris* (dog), and *Gallus gallus* (chicken).

Finally, the enrichR library offers functional enrichment for seven organisms, namely *Homo sapiens* (human), *Mus musculus* (mouse), *Bos taurus* (bovine), *Drosophila melanogaster* (fruit fly), *Caenorhabditis elegans* (nematode worm), *Saccharomyces cerevisiae* (yeast), and *Danio rerio* (zebrafish), preferably in the Entrez gene namespace and supports extra enrichment options for phenotypes through the KOMP2 Mouse Phenotypes, WormBase (Davis *et al.* 2022), and MGI Mammalian Phenotype (Ringwald *et al.* 2022) databases.

Through Flame, users can perform enrichment using each individual tool, or can select to combine multiple tools. The combination of tools that can be used depends on their supporting the organism chosen for the analysis. Enrichment can be performed using the entire organism’s genome as the reference with any of the four tools; alternatively, users can also upload a custom reference background of their own and perform enrichment with aGOtool, g: Profiler, and WebGestalt.

A summary and a direct comparison of the supported features, cutoff types and allowed namespaces are shown in Table 1. Regarding execution times, aGOtool is the default selected enrichment tool of Flame and performs the fastest while WebGestalt’s API executes the slowest and may affect Flame’s performance.

**Table 1.** Comparison of the four different enrichment tools integrated in Flame.

	aGOtool	g: Profiler	WebGestalt	enrichR
Organisms	14 436	821	12	7
Namespaces	Ensembl IDs, UniProt ACs, STRING IDs	Ensembl, Entrez, UniProt, RefSeq, EMBL, ChEMBL, WikiGene	Entrez gene accession identifiers	Entrez gene names
Significance metrics	*P*-value, false discovery rate (FDR)	g: SCS (adjusted *P*-value), FDR, Bonferroni	Bonferroni and five FDR types (BH, BY, Holm, Hochberg, Hommel)	Adjusted *P*-value
Option to use custom background	Yes	Yes	Yes	No
Supported databases				
Gene ontology	Biological processes, molecular functions, cellular components	Biological processes, molecular functions, cellular components	Biological processes, molecular functions, cellular components	Biological processes, molecular functions, cellular components
Biological pathways	KEGG, REACTOME, WikiPathways	KEGG, REACTOME, WikiPathways	KEGG, REACTOME, WikiPathways, PANTHER	KEGG, REACTOME, WikiPathways,
				PANTHER
Diseases	Disease ontology	N/A	OMIM, DisGeNET, GLAD4U	Disease ontology
Drugs	N/A	N/A	DrugBank, GLAD4U	N/A
Protein families, domains and complexes	InterPro, Pfam, UniProt	CORUM	N/A	N/A
Tissues	BRENDA tissue ontology	Human protein atlas	N/A	WormBase
Phenotypes	N/A	Human phenotype ontology (HPO)	Human phenotype ontology (HPO)	Human phenotype ontology (HPO), KOMP2 mouse phenotypes, WormBase, MGI mammalian phenotype
Regulatory DNA motifs	N/A	TRANSFAC, miRTarBase	N/A	N/A

### 2.3 Visualization options

**Figure 2.**
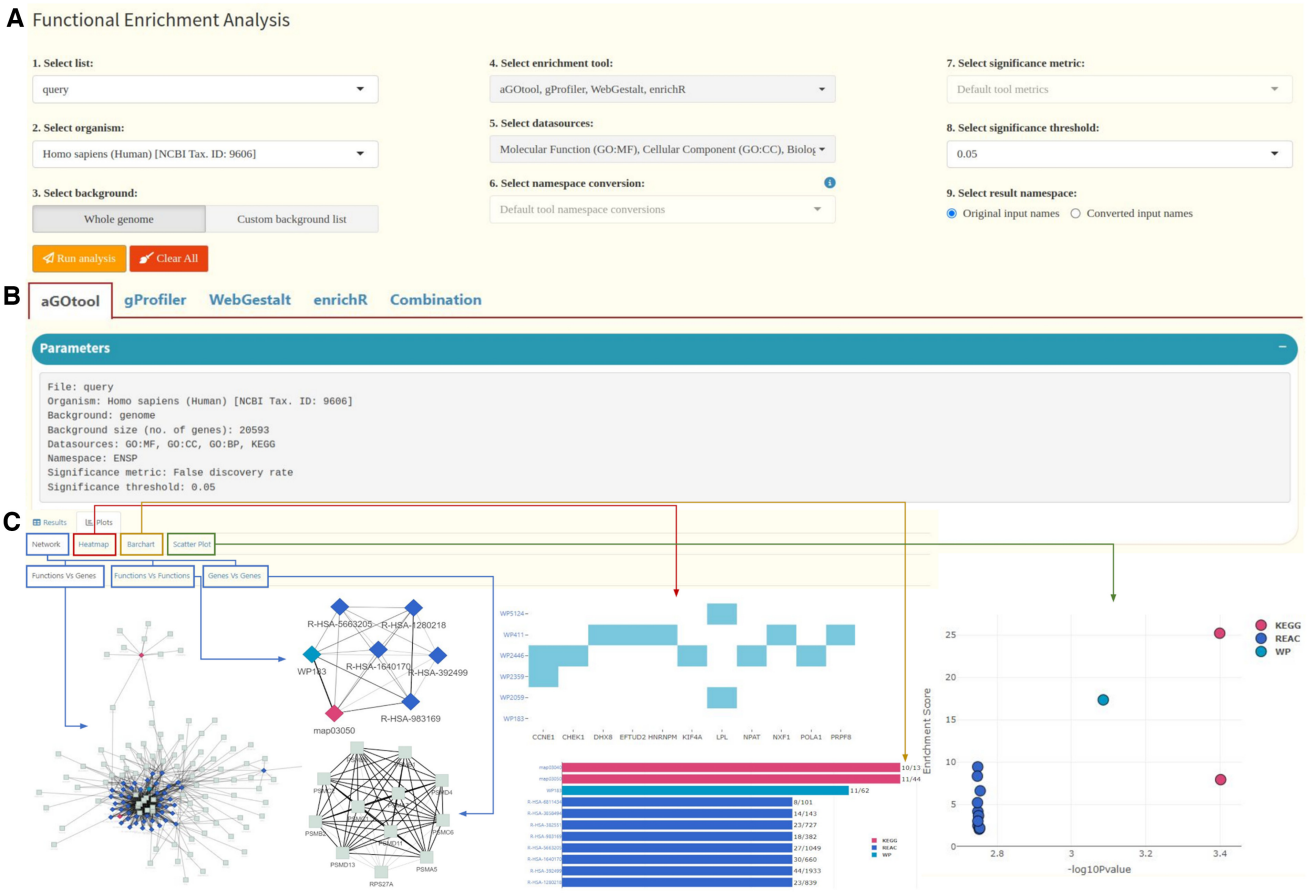
Summary of Flame’s functionality. (A) Selection of list, organism, reference background, enrichment tool(s), datasources, namespace, significance metric, and threshold. (B) aGOtool, g: Profiler, WebGestalt and enrichR results are reported on separate tabs as well as unified into a tab called “Combination”. (C) Visualization options for reporting the top enriched terms (networks, heatmaps, bar charts and scatter plots). Networks and heatmaps can be used to show *function-gene*, *function-function* and *gene-gene* associations while top hits from multiple sources can be shown in combination as a multi-type network, bar chart or scatter plot

Flame extends its functionality by offering a plethora of visualizations and network analysis options for the interpretation of enrichment results. Besides simple searchable and interactive tables, it offers several visual alternatives for presenting the reported results from each enrichment tool (Fig. 2). Flame provides four different main visualization options to represent the top enriched terms. These are: (i) networks, (ii) heatmaps, (iii) barcharts and (i) scatter plots. For each category, one can adjust the top hits interactively by setting up filters and parameters such as datasources, number of visualized top terms and scoring metric. In the case of networks and heatmaps, three different modes of association are offered, namely *Functions* versus *Genes, Functions* versus *Functions*, and *Genes* versus *Genes*. In the first mode, the top functions and their associated genes are presented either as a network (nodes linked with edges), or as a heatmap (associations are shown as colored cells/blocks). In the second mode, functions are connected with other functions based on the number of their shared genes, whereas in the third mode, genes are connected based on the common functions they are involved in. In the cases of networks, bar charts and scatter plots, one can adjust the top hits by combining multiple types of resources (e.g. pathways from KEGG, Reactome and WikiPathways). In addition, while most of the network associations are shown in 2D, Flame has the option to call Arena3D^web^ (Karatzas *et al.* 2021, Kokoli *et al.* 2023) in order to visualize heterogeneous information in a 3D view as a multi-layered graph (each biomedical entity type corresponding to a different layer).

All aforementioned plots are also available for the literature enrichment pipeline, where instead of functions, PubMed articles are returned. A Manhattan plot can also be generated for g: Profiler exclusively. Finally, as an extra analysis step, Flame utilizes the STRING API to generate protein-protein interaction networks, which can subsequently be used to also perform enrichment analysis.

### 2.4 Use of UpSet plots for comparing input lists and reported results from various sources

**Figure 3.**
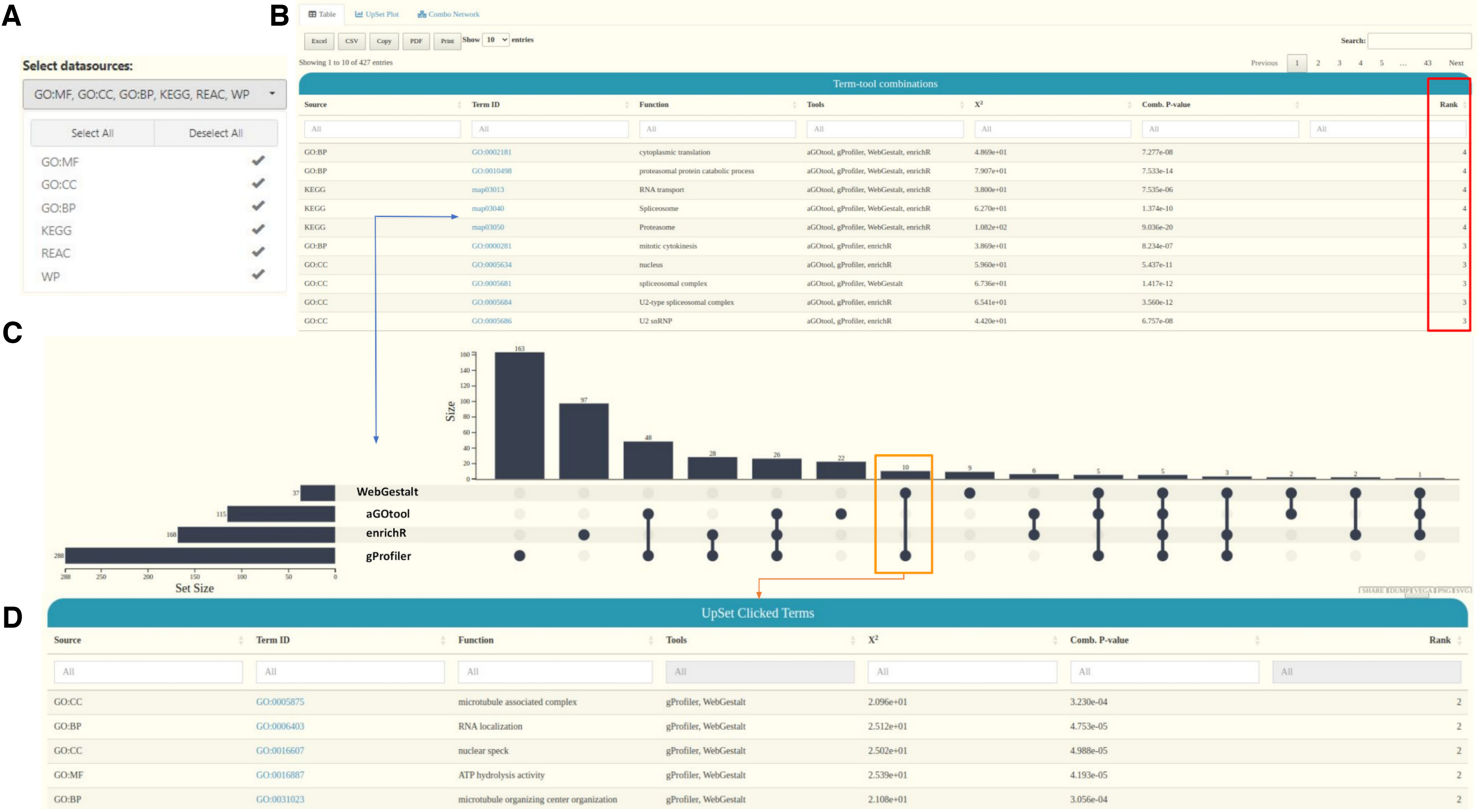
Distinct intersections of reported results. (A) A selection box to limit the comparisons to certain biomedical entities. (B) A searchable and interactive table reporting the intersecting biomedical entries from each tool. (C) A visual representation of the distinct intersections with the use of interactive Upset plots. (D) Part of a table showing 5 of the 10 biomedical entries of the distinct intersection set which corresponds to the chosen UpSet bar

Flame (v1.0) uses UpSet plots, as a replacement for Venn diagrams, in order to show unions, intersections and distinct combinations among the input gene lists. Following the same approach, in this version, Flame (v2.0) extends this functionality to show how the four supported back-end pipelines (*aGOtool, gProfiler, WebGestalt, enrichR*) agree on the results they report (Fig. 3).

To this end, when more than one functional enrichment tool has been executed, an extra tab named “*Combination*” is generated automatically. In this tab, distinct intersections between the four resources are summarized in a searchable and easy-to-filter table as well as in an interactive UpSet plot. The table reports the aggregated enriched terms along with the tool(s) that generated the term and its respective rank (number of tools that returned it). Rank 1 means that a biomedical entity (e.g. an enriched pathway) was detected by one resource only, while rank 4 means that all four tools fetched the same result. In addition to the above, the statistical significance of the combined results is estimated by applying Fisher’s combined probability test (Mosteller and Fisher 1948), represented by *X*^2^ and combined *P*-value metrics for each enrichment term identified by two or more tools.

In parallel, Flame (v2.0) generates an interactive UpSet plot where users can click on a bar to generate a table which corresponds to the subset of shared terms shown in the corresponding UpSet column. In this implementation, the UpSet plot consists of two to four rows (one for each enrichment tool) while columns describe the distinct intersections among results. Both the table and the UpSet plot can be limited to a user-selected set of biomedical entries (e.g. compare reported pathways, or biological processes or both). In addition, Flame offers the option to visualize the combined results as a *Functions* versus *Genes* association network (as described in the previous section), in which the weight of the edges corresponds to the number of tools supporting the corresponding gene–function associations.

### 2.5 Implementation

Flame is mainly written in R (Shiny framework). The enrichment analysis with aGOtool is executed through its corresponding API (https://agotool.org/api_orig). Enrichment analysis with g: Profiler, WebGestalt and Enrichr is executed through their R libraries; *gprofiler2*, *WebGestaltR*, and *enrichR* respectively. Additional statistical calculations are performed using the *PoolR* package (Cinar and Viechtbauer 2022). Enriched term networks are generated through the *igraph* R package and visualized via the *visNetwork* R library. The volcano plot input along with all other enriched term plots (heatmaps, barcharts and scatter plots) are generated with the use of the *plotly* R library. A Manhattan plot is also offered, specifically for the g: Profiler results, through its respective package. Finally, UpSet plots are drawn with the use of *upsetjs* R library.

The SNP input and namespace/orthology conversions are handled through the g: Profiler package. Text-mining input is handled by the EXTRACT tagger API (https://tagger.jensenlab.org). The PPI network analysis is offered by the STRING API (https://string-db.org/api). Flame handles its GET API requests through R and its POST API requests through a dedicated node.js server.

### 2.6 Application programming interface

Flame comes with its own API and currently supports both GET and POST requests. Shorter requests can be done with a GET request using the following format:

https://bib.fleming.gr:8084/app/flame/?url_genes=MCL1,TTR;APOE,ACE2;TLR4,HMOX1

In this example, gene lists are separated with semicolon (;) and genes with comma (,). Longer requests can be performed via Flame’s POST request at:

https://bib.fleming.gr/bib/api/flame

using the following JSON object format:

{"gene_list1": ["GNAS", "ABCG2", "WT1", "CDK2", "FLT1", "HBA1", "CCN2", "MDM2"],

"gene_list2": ["MMP1", "PTGS2", "PON1", "LDLR", "HBA1", "CYP1B1", "PTEN", "SNCA"],

"gene_list3": ["UGT1A1", "CDH1", "MDM2", "EGFR", "FMR1", "VEGFA", "ERCC1"]}

## 3 Discussion

Flame (v2.0) expands on its previous capabilities in many aspects (Table 2); from offering users a plethora of different input options, to a significant organism-space expansion, the addition of multiple enrichment libraries/APIs and the combination of their results, as well as through enhancing its own API. We believe that the multiple input options along with STRING’s organisms, make Flame a very competitive application which greatly enhances user experience. By also offering the streamlined execution of multiple functional enrichment tools and the aggregation of their results, we facilitate the knowledge extraction of analysis, and partially tackle the question “which tool should I use/trust?”. Given its accessibility and ease of use, we hope Flame will quickly become the go-to tool for advanced functional enrichment analysis and visualization.

**Table 2.** Main differences between Flame versions.

	Flame (v1.0)	Flame (v2.0)
Enrichment categories	Gene ontology Biological pathways Proteins Phenotypes Tissues Regulatory motifs Literature enrichment	Gene ontology Biological pathways Proteins Phenotypes Tissues Regulatory motifs Literature enrichment Drug associations Protein families Diseases
Enrichment tools	g: Profiler aGOtool (only for literature enrichment)	aGOtool g: Profiler WebGestalt enrichR
Supported organisms	197	14 436
Input options	Gene/identifier lists	Gene/identifier lists SNP lists NER and text-mining on a free text Gene selection from an interactive volcano plot Support of custom background lists
List comparison with the use of UpsetPlots	Input gene lists	input gene lists reported results from any of the four supported tools
Other types of analysis	Protein–protein interaction networks Gene orthology search Database id conversion	Protein–protein interaction networks Gene orthology search Database id conversion Mapping of SNPs to genes Statistical testing when combining multiple *P*-values from various tools Functional and literature enrichment for the generated STRING PPI network
Visualization	Plots (Bar, UpSet, Scatter, Manhattan) Heatmaps 2D Networks with the use of VisNetwork	Plots (Bar, UpSet, Scatter, Manhattan) Heatmaps 2D Networks with the use of VisNetwork Gene–function network visualization for entities from multiple sources 3D multilayer graphs with Arena3D^web^
API requests	GET	GET POST

## Data Availability

The software and data underlying this article are available at https://github.com/PavlopoulosLab/Flame.
